# On the Practical Study of Botany, by Means of Chemical Analysis: Chiefly Abridged from the Original Papers of Dr. T. S. Hermbstaedt, &c.

**Published:** 1799-05

**Authors:** 


### *On the practical Study of Botany, by means of chemical Analyſis: Chiefly abridged from the original Papers of Dr.* T. S. HERMBSTAEDT, *&c.*

[Continued from No. II. p. 159---165.]

IN the chemical analyſis of a vegetable ſubſtance, we propoſe either to inquire into the nature of its formative elements, or to diſcover the cauſes of its medicinal virtues. In the former view, the greater number of vegetable bodies diſplay a remarkable coincidence or ſimilarity in thoſe conſtituent parts which we are enabled to exhibit in a ſeparate ſtate: while thoſe, the knowledge of whoſe reſpective properties, in a diſtinct form, would be of the greateſt advantage, but rarely admit of being ſeparately exhibited. With regard to the latter caſe, the chemical analyſis of a vegetable ſubſtance cannot afford us any inſtruction, concerning the capacity of that ſubſtance to produce poſitive effects in the animal body; and were we to undertake the analyſation of organic bodies with that intention, it would be productive of no advantage. Notwithſtanding theſe reſtrictions, the chemical analyſis of ſuch a ſubſtance, if undertaken according to determinate principles, may pave a ſure way to the diſcovery of thoſe elements, on which its medicinal effect principally depends; and, in this view, our chemical experiments on vegetables muſt be attended with obvious advantage to the therapeutical practitioner.

If we diſcover the ſpecific power of a plant, or any particular part of it, ſuch as the root, the leaf, the flower, the wood, the bark, the fruit, &c. we always find ourſelves indebted, for this diſcovery, to the uſe of it in ſubſtance. The reflecting medical practitioner will, upon this occaſion, ever inquire, what principle gave this plant its medicinal power? was it really a particular conſtituent conftituent part of it, or was it the whole plant, or the whole of its fubftance, which produced that effect? The difcovery of this circumftance is naturally the firft and moft important point of view, in which the chemift attempts a fcientific analyfis of plants, with refpect to their medicinal properties.

We may confider every plant, or its particular parts, as a compound of various heterogeneous conftituents. We fhall find that the whole of its active powers were determined either by a particular conftituent part of it, or that its virtue depended on a combination of two different conftituents; or laftly, on the combined activity of all its elementary parts. The ufual procefs formerly adopted by chemifts, was that of afcertaining the proportion of watery, oleaginous, or pure gummy parts contained in fuch a plant, and the particular effence or tincture which it afforded. Such a chemical analyfis muft naturally have been very fallacious, and altogether unfit for illuftrating the medicinal powers of plants; as in this manner the greater number of them could not exhibit any remarkable difference.

A true chemical decompofition of a vegetable body, on the contrary, will prove, that fuch a body is neceffarily compounded of a great variety of heterogeneous ingredients, which, by chemical procefs, can be feparated from each other, and exhibited as diftinct fubftances. If we fucceed in this attempt, fo that none of the different conftituents is thereby changed in its nature, its efficient power muft confequently remain unaltered. If, in this manner, we fhall reduce a vegetable body to its proximate elementary parts, that is, fuch as are not fubject to any farther change, then only are we enabled to afcertain with plaufibility the medicinal effects of each individual conftituent part. Should it therefore be found, that one of thefe parts is capable of producing that effect, which we have formerly obferved to take place from the ufe of the whole plant in its integral ftate, we may juftly conclude, that this part alone contains its medicinal virtue. If, however, it fhould be obferved, that none of the individual conftituents manifeft this power, it will then be neceffary to recur to experiment, whether a combination of two, three, or more of them, previoufly difcovered, or all of them jointly, are capable of producing that effect.—After having thus learned by experience the knowledge of that fubftance which is the principal agent in a particular plant, we fhall then be enabled to judge, what medicines may be prepared from it, and what chemical procefs ought to be adopted in fuch preparation. This is the only true point of view in which the chemical analyfis of a plant fhould be undertaken, if the inquirer expects to improve the Materia Medica by fuch purfuits.

---

An accurate examination of vegetable bodies will readily inform us, that thofe elements, of which naturehas compofed them, may be aptly divided into par parts mixed in an *aggregate form*, and into parts *chemically combined*. Among the former are comprehended those vegetable ingredients, the presence of which may be discovered without recurring to chemical analysis, which consequently can be exhibited as distinct objects, without a complete decomposition of the elementary mixture of such vegetables. Of this nature are, 1. water; 2. the saccharine ingredient; 3. the basis of oil; 4. that of camphor; 5. fat, &c. All those parts, on the contrary, which are so intimately blended, that they can only be recognized and exhibited after a complete dissolution of the elementary mixture, justly deserve to be denominated *constituent* parts, or elements chemically combined.

From the tables of elementary constituents already exhibited*, in which the primary, as well as the secondary elements of vegetable bodies, together with the simple vegetable acids, have been enumerated, it it is obvious, that such bodies contain a considerable number of heterogeneous ingredients. It is hence also self-evident, that a chemical analysis of this kind, to be rendered productive of real utility, will require an uncommon degree of accuracy, and indefatigable perseverance.

As it is not in every instance, even with the most experienced chemical inquirer, an easy matter to determine, to what particular constituent parts he ought to direct his attention, in analysing a vegetable body; it appears to be equally useful and necessary, to specify some chemical tests, or re-agents, by means of which he may previously ascertain the general constituent parts of any particular plant, with a certainty similar to that with which he has long been accustomed to ascertain those of mineral waters.

The following is a list of the chemical re-agents hitherto used by Dr. Hermbstaedt, the number of which, however, might be considerably increased:—

1. A perfectly pure *alkohol*.

2. A very pure *vitriolic ether*, which is freed from water, as well as spirit of wine.

3. Pure *distilled* water.

4. A somewhat concentrated solution of the *acetite of barytes*.

5. A solution of the *muriat of lime*.

6. A saturated solution of the *muriat of iron*, which ought to be afterwards diluted with water.

7. A solution of the *sulphat of silver*.

8. A highly *concentrated acetic acid*.

9. Rectified

* See No. I. p. 75, and No. II. pp. 159, 162.

9. Rectified *petroleum*, or rock-oil.

10. A concentrated ſolution of pure kali or potaſs, or *cauſtic vegetable alkali*.

11. A ſolution of the *acetite of lead*.

12. *Blue turnſol paper*, or *litmus*, properly prepared.

13. *Cauſtic ſpirit of ſal ammoniac*, or *aqua ammoniæ puræ*.

14. *Carbonat of potaſs*, or *kali præparatum in cryſtals*.

15. A very pure *nitric acid;* and—

16. A *concentrated vitriolic acid*.

*Of the Method of employing theſe Re-agents.*

In order previouſly to examine, by means of theſe re-agents, the conſtituent parts of a particular vegetable body, we ought to procure ſmall glaſs retorts, containing about one ounce meaſure. Such veſſels are convenient, not only for making experiments, on a ſmall ſcale, with vegetable bodies, but likewiſe with any other ſubſtances. With ſuch an apparatus, and the neceſſary re-agents before enumerated, we may confidently begin our experimental inquiries in the manner following:—

A. To aſcertain the preſence of *reſinous matter*, a ſmall quantity of the powder of the vegetable ſubſtance under experiment is placed in a glaſs retort, with three times its weight of vitriolic ether; the whole is allowed to ſtand for half an hour in the open air, if in ſummer, or in a moderately warm room, if in winter, occaſionally ſhaking the veſſel. If during that time, the ether ſwimming on the top has not changed its colour, it is a certain criterion that no reſinous particles are contained in the ſubſtance under examination. On the contrary, as the ether is more or leſs tinged, the quantity preſent of the reſinous matter may be inferred.

B. The *ſaponaceous* principle may be eaſily diſcovered in ſubſtances yielding no reſinous parts, by pouring on another ſmall quantity, in the retort, three times the weight of alkohol, treating it in a manner ſimilar to the preceding, and, if neceſſary, ſomewhat increaſing the degree of heat. If the alkohol remain untinged, the vegetable contains no ſaponaceous matters, as theſe would be held in ſolution by the alkohol. According to another proceſs, the examination of a ſubſtance, as to its reſinous and ſaponaceous ingredients, may be undertaken at one and the ſame time. As alkohol is capable of diſſolving both ingredients, the vegetable body only requires to be digeſted in that liquid. If after this proceſs, the clear ſolution, being poured into ſix times the quantity of cold water, become turbid, the preſence of reſin is then manifeſt; but if no turbidneſs ariſe, it contained only ſaponaceous matter.

C. To

C. To prove the exiſtence of *ſaccharine* matter, we ought to attend to the following circumſtance. If by the preceding teſt the preſence of ſaponaceous matter has been already diſcovered, the ſame vegetable body may be ſubjected to the teſt of alkohol, in a more elevated temperature. Thus the ſaccharine ingredient will be likewiſe ſeparated, and may be afterwards obtained by cryſtalliſing the ſugar in a reduced temperature.

D. The matter of *gum* may be ſeparated from vegetable ſubſtances, by extraction, in a moderate heat, with diſtilled water. Should the ſolution be turbid, which would indicate, that it is alſo mixed with reſinous particles, it ought to ſtand till it become cool, and then be filtered through porous paper: the clear fluid muſt next be evaporated until, in a cold place, it acquire the conſiſtency of a thin ſyrup. In this ſtate, three parts of alkohol, expoſed to a reduced temperature, muſt be poured upon it; and if a complete ſolution take place, we may then conclude, that the whole of it conſiſted of pure matter of ſoap, without any particles of gum; for, if the latter had been preſent, it would have been ſeparated from this fluid in tender flakes.

E. The preſence of *mucilage* can be aſcertained in a manner analogous to the preceding, as the ſame ſolvents are applicable to both. But in order to diſtinguiſh it, with ſufficient accuracy, from the ſubſtance of gum, as has already been pointed out, (No. I. Page 75, of this Journal,) it is indiſpenſably neceſſary to attend to the following particulars: whether the ſeparated matter be ſlippery between the fingers, or whether it can be drawn in threads. Beſides, the preſence of mucilaginous matter in ſome bodies, ſuch as the orchis, marſhmallows, &c. may be readily diſcovered from their farinaceous nature, as well as by gelatinous ſolutions of them, made in ſimple water.

F. The preſence of *albuminous* matter in vegetables, may be aſcertained in two different ways, according as they are in a *freſh* or *dried* ſtate. In the former it is only neceſſary to bruiſe a ſmall quantity of the ſubſtance under experiment, to expreſs the ſap, to filter it through paper, and to divide the clear liquid into two parts: one of theſe parts is to be mixed with half its quantity of alkohol, and if no turbidneſs or precipitation take place, we may be convinced, that it contains neither matters of gum, mucilage, nor albumen. But, if the liquor ſhould become turbid, this circumſtance may indicate the preſence of either albuminous, gummy, or mucilaginous ingredients. In this caſe, the ſecond portion of the clarified ſap ought to be heated to the boiling point; and if it contain any albumen, it will be eparated in ſmall flakes, reſembling thoſe formed by boiling the white of eggs eggs in water to a hard consistence, and emitting the smell of burnt horn. If on the contrary, dried vegetables be the subject of experiment, the substance ought to be previously extracted by means of alkohol, and afterwards by the conjoined agency of water and heat. Should ammoniacal or other neutral salts be contained in them, they will pass over into the latter solution. If the substance should contain albuminous matter, this may be discovered from the viscid and elastic nature of the vegetable fibre which forms the residuum: in a perfectly dry state, this residuum, if placed on red-hot coals, acquires a hard horny form, and emits the empyreumatic smell of horn.

G. If we surmise the existence of *elastic gum* as a part of a vegetable body, it ought to be treated in the manner before mentioned, first, by alkohol, and then by water; the residuum ought afterwards to be well digested in double its quantity of rectified *petroleum*, or of *vitriolic ether*. This solution must be mixed with an equal part of alkohol; in that state it either remains unchanged, or a precipitate is formed, consisting of a viscid, greasy substance, which takes fire upon being exposed to a candle, and emits the smell of burnt bacon; in this latter case, the inflammable substance is the elastic resin, the presence of which was designed to be discovered by the experiment.

H. The matter of *wax*, as forming an ingredient in many vegetables, is discoverable, partly from their shining surface, partly from a certain flexibility in such bodies. In order to ascertain its presence, a sufficient quantity of the substance under experiment ought to be infused in four times its weight of water of pure ammonia, or *caustic spirit of sal-ammoniac*, and kept for some time in a state of digestion. After sufficient maceration, the liquor is carefully expressed, filtered, and mixed with such a quantity of distilled vinegar, as may render it a predominant ingredient of the compound. Thus a pale yellow powder is precipitated, which frequently consists of a combination of waxy and resinous particles. If this powder be infused in alkohol, the resinous parts will be completely dissolved, while those of wax remain behind.

I. The *astringent principle* may be readily discovered. A few drops of a spirituous or watery extract of a vegetable substance, should be poured into a glass vessel, containing distilled water: to this mixture a drop or two of a solution of the muriat of iron, should be added and stirred up with it. By this addition the astringent principle will immediately shew itself, by changing the colour of the mixture to a black or reddish brown.

K. Although the *acrid* or *pungent principle*, cannot always be discovered by the taste and flavour of vegetable substances, yet it is not difficult to determine mine the preſence of that principle, by diſtilling them in ſimple water. The product will thus manifeſt a conſiderable degree of pungency: and, in this manner, Profeſſor Hermbſtaedt was enabled to exhibit to the ſenſes the pungent principle, as a conſtituent even of the leſſer centaury. (*Gentiana centaurium, Linn.*)

(To be continued in the next Number.)

